# Impact of Long-Term Treatment with Ivermectin on the Prevalence and Intensity of Soil-Transmitted Helminth Infections

**DOI:** 10.1371/journal.pntd.0000293

**Published:** 2008-09-10

**Authors:** Ana Lucia Moncayo, Maritza Vaca, Leila Amorim, Alejandro Rodriguez, Silvia Erazo, Gisela Oviedo, Isabel Quinzo, Margarita Padilla, Martha Chico, Raquel Lovato, Eduardo Gomez, Mauricio L. Barreto, Philip J. Cooper

**Affiliations:** 1 Instituto de Microbiologia, Universidad San Francisco de Quito, Diego Robles y Via Interoceanica S/N, Cumbaya, Quito, Ecuador; 2 Instituto de Saude Colectiva, Universidad Federal da Bahia, Rua Basílio da Gama, s/n - Campus Universitário Canela, Salvador, Bahia, Brazil; 3 Instituto de Matemática, Universidad Federal da Bahia, Instituto de Matemática - Campus de Ondina, Ondina, Salvador, Bahia, Brazil; 4 Programa Nacional de Eliminacion de Oncocercosis del Ecuador, SNEM, Guayaquil, Ecuador; 5 Centre for Infection, St George's University of London, Cranmer Terrace, Tooting, London, United Kingdom; George Washington University, United States of America

## Abstract

**Background:**

Control of soil-transmitted helminth (STH) infections relies on the periodic and long-term administration of anthelmintic drugs to high-risk groups, particularly school-age children living in endemic areas. There is limited data on the effectiveness of long-term periodic anthelmintic treatment on the prevalence of STHs, particularly from operational programmes. The current study investigated the impact of 15 to 17 years of treatment with the broad-spectrum anthelmintic ivermectin, used for the control of onchocerciasis, on STH prevalence and intensity in school-age and pre-school children.

**Methods and Findings:**

A cross-sectional study was conducted in communities that had received annual or twice-annual ivermectin treatments and geographically adjacent communities that had not received treatment in two districts of Esmeraldas Province in Ecuador. Stool samples were collected from school-age children and examined for STH infection using the Kato-Katz and formol-ether concentration methods. Samples were collected also from pre-school children and examined by the formol-ether concentration method. Data on risk factors for STH infection were collected by parental questionnaire. We sampled a total of 3,705 school-age children (6–16 years) from 31 treated and 27 non-treated communities, and 1,701 pre-school children aged 0–5 years from 18 treated and 18 non-treated communities. Among school-age children, ivermectin treatment had significant effects on the prevalence (adjusted OR =  0.06, 95% CI 0.03–0.14) and intensity of *Trichuris trichiura* infection (adjusted RR = 0.28, 95% CI 0.11–0.70), but appeared to have no impact on *Ascaris lumbricoides* or hookworm infection. Reduced prevalence and intensities of *T. trichiura* infection were observed among children not eligible to receive ivermectina, providing some evidence of reduced transmission of *T. trichiura* infection in communities receiving mass ivermectin treatments.

**Conclusion:**

Annual and twice-annual treatments with ivermectin over a period of up to 17 years may have had a significant impact on *T. trichiura* infection. The present data indicate that the long-term control of onchocerciasis with ivermectin may provide additional health benefits by reducing infections with trichuriasis. The addition of a second anthelmintic drug such as albendazole may be useful for a long-term effect on *A. lumbricoides* infection.

## Author Summary

Soil-transmitted helminth (intestinal worm) infections are very common in developing countries and are an important cause of illness. Mass de-worming treatments of school children are an important strategy to reduce illness caused by these infections in communities without access to clean water and sanitation. Few studies have examined the effect of repeated mass treatments in the long-term in controlling these infections. The objective of the present study was to assess the impact of the drug ivermectin used for the control of onchocerciasis (river blindness), that has important effects against intestinal worms, on the epidemiology of intestinal worms in children when administered repeatedly for 15–17 years. We compared the epidemiology of infections between children living in communities that received ivermectin with communities that never received the drug. The data suggest that ivermectin has important differential effects on intestinal worms with a greater impact on infections with *Trichuris trichiura* and little impact on *Ascaris lumbricoides* and hookworms infections. Our data suggest that long-term ivermectin treatments may provide health benefits through effects on *T. trichiura* infections but that the addition of second de-worming drug such as albendazole may be required for the control of other intestinal worm infections.

## Introduction

Soil-transmitted helminths (STHs, also known as geohelminths or intestinal helminths) are important infectious diseases of humans and are estimated to infect over 2 billion humans worldwide [1], being particularly prevalent in poor populations living in tropical and sub-tropical regions of developing countries [2]. Soil-transmitted helminths include *Ascaris lumbricoides*, *Trichuris trichiura*, hookworm, and *Strongyloides stercoralis*, and are considered to be a major cause of morbidity related to impaired nutrition and poor childhood growth [3],[4].

STH control strategies are focused currently on mass treatment with broad spectrum anthelminthic drugs with the aim of reducing morbidity through reductions in parasite burdens that are strongly associated with risk of morbidity [5],[6]. Because of a high risk of re-infection in endemic areas, treatments have to be administered repeatedly for long periods of time. WHO has recommended the use of one of albendazole, mebendazole, levamisole and pyrantel for STH control [7]. The 4 drugs have variable efficacy against different STH parasites.

Ivermectin is a broad-spectrum anthelmintic drug used for the control of filarial infections including onchocerciasis and lymphatic filariasis [8],[9]. Ivermectin has also efficacy against STH infections - it is highly effective against ascariasis and strongyloidiasis [10],[11],[12],[13],[14] for which it has superior or comparable efficacy to albendazole [10],[12], is of moderate efficacy against trichuriasis for which it has comparable efficacy to albendazole [10],[11],[13],[14] but is less effective than albendazole against hookworm [12],[13],[14].

The distribution of ivermectin forms the basis of onchocerciasis control programmes worldwide. In Ecuador, the National Programme for the Elimination of Onchocerciasis has administered annual or twice-annual mass treatments with ivermectin over the past 17 year as the primary control strategy in endemic communities and has achieved high rates of coverage. We have reported previously that ivermectin control has been extremely effective for the control of onchocerciasis and may have eliminated the infection from some infection foci in Ecuador [15].

To investigate the long-term effect of ivermectin treatment used for the control of onchocerciasis on the prevalence of infection and transmission with STH parasites, we compared the prevalence and intensities of STH infections among children living in communities that had received long-term ivermectin treatments with those that had not received ivermectin in Esmeraldas Province in Ecuador. Previous surveys conducted in this area have shown high rates of STH infection [16],[17].

## Methods

### Study area and population

This study was conducted in rural Afro-Ecuadorian communities located in the principal onchocerciasis focus or in adjacent communities in the districts of Eloy Alfaro and San Lorenzo in Esmeraldas province, in northeastern Ecuador. The area is equatorial rain forest at an altitude of below 100 m above sea level and the characteristics of the study area and population are described in detail elsewhere [18].

### Study design

A cross-sectional study was conducted to compare the prevalence and intensity of STH infections between children from treated and non-treated Afro-Ecuadorian communities. Selection of ivermectin-treated study communities was based on the 6-monthly treatment schedule with ivermectin of the Ecuadorian Elimination Programme for Onchocerciasis. Non-treated communities with a recent census were selected to be as similar as possible to treated communities with respect to geographic location (non-treated communities were geographically close to treated communities within the 2 study Districts), size (communities with schools having >250 pupils were excluded), and similar economic activities (e.g. subsistence agriculture, logging, and hunting). None of the non-treated communities had received previous mass treatments with ivermectin. STH infections were examined in two distinct age groups using the community census: 1) school-age (6–16 years) including children attending and not attending community schools; and 2) pre-school (0–5 years). These age groups separated approximately children eligible to receive ivermectin (>15 kg) from those not eligible, allowing an assessment of the effect of ivermectin treatments on the transmission of STH infections. Assessment of pre-school children was performed for a sample in the treated and non-treated communities.

### Data collection

The field study was conducted between March 2005 and May 2007. Stool collections were performed immediately before 6-monthly ivermectin distributions in treated communities. Annually-updated censuses were used to identify eligible children. A register of the number of treatments received by each child was obtained from the National Programme for the Elimination of Onchocerciasis. A questionnaire was administered to the parent or guardian of each child by trained field workers to obtain information about risk factors for STH infections including age, sex, mother's education level, material goods in the household, household income, household crowding, access to water, sanitation and recent anthelmintic treatments [18].

### Examination of stool samples

Single stool samples were collected from each child and examined for STH eggs and larvae using the modified Kato-Katz technique (quantification of *Ascaris lumbricoides* and *Trichuris trichiura* infection) [school-age children only] and formol-ethyl acetate concentration (detection of all STH infections including hookworm and *Strongyloides stercoralis*) [19] [all children].

### Distribution of ivermectin

The National Program for Elimination of Onchocerciasis has used community-based mass distribution of ivermectin as the main strategy for the control of *Onchocerca volvulus* infection since 1990. There are a total of 119 endemic communities with an estimated 19,420 inhabitants in 2 Provinces in Ecuador and ivermectin treatment was gradually introduced into all communities over the period 1990–1997. The number of treatments and the start of mass distribution vary according to each focus. Mass treatment was initiated in all communities included in this study between 1990 and 1992, and the period of mass ivermectin treatments for study communities ranged 15–17 years. Initially, ivermectin was distributed annually to endemic communities but treatments were increased to 6-monthly in all communities from 2001. Treatment coverage with ivermectin has been high and over the period 1990 to 2003, the annual average treatment coverage per treatment round with ivermectin was 85.2% (range 54.9%–97.9%). The mean treatment coverage for the two years, 2005–2006, was 98%. Eligibility criteria for treatment are: weight greater than 15 kg and free of serious illness (e.g. active tuberculosis, terminal cancer etc), and for women, not pregnant and not nursing infants up to 3 month of age. Ivermectin is distributed by primary health workers in each community at a dose of 150 µg/mg and treatments are directly observed. The population census is used as the basis for drug distribution [15].

### Statistical analysis

STH prevalence was expressed as the percentage of subjects found positive for each parasite. Comparisons between proportions were performed using an adjusted chi-squared test for clustered data. ORs for prevalence data were calculated using multilevel logistic regression models (command, xtlogit) to obtain robust standard errors that take into account correlated data. Potential confounding factors were controlled for in the analysis. A multilevel analysis was used because of the large variation in STH prevalence between study communities. The intraclass correlation coefficients for *Ascaris* and *Trichuris* infection in this study were 0.29 (95% CI: 0.21–0.39) and 0.13 (95% CI: 0.09–0.18), respectively.

STH intensity was calculated as eggs per gram of stool. Because stool egg counts were over-dispersed (*A. lumbricoides*, mean 6,297 epg, variance 5.1×10^8^; *T. trichiura*, mean 9746 epg, variance 1.3×10^7^), a zero-inflated negative binomial (ZINB) model was used to fit the data. The Vuong test (1.90 (p = 0.03) and 11.55 (p<0.001) for *A. lumbricoides* and *T. trichiura*, respectively) indicated that a zero inflated model fit the data better than a standard negative binomial model [20]. A robust variance estimator was used to account for clustering by community, and rate ratios (RRs) and their 95% confidence intervals were estimated. The ZINB model was fitted manually, and potential confounding factors were controlled for in multivariate analyses. RRs for infection intensity indicate the relative increase or decrease in infection intensities in the treated compared to non-treated groups. A single 0.5 unit fall in RR represents a 50% greater infection intensity in the untreated compared to treated group. All analyses were done with STATA (version 9.0).

### Ethics

The study protocol was approved by ethical committee of the Hospital Pedro Vicente Maldonado, Pichincha Province, Ecuador. Informed written consent was obtained by a parent or guardian for all children. All children were offered treatment with a single dose of 400 mg of albendazole at the end of the study.

## Results

### Study population and anthelmintic treatment

A total of 3,960 school-age children from 58 communities were assessed and 3,705 provided a stool sample and were included in the analysis. School-age children were analysed from 31 (1,752 children) ivermectin-treated and 27 (1,953 children) neighbouring non-treated communities. The mean cluster size was 54.7 (range: 13–203) children in treated and 69.2 (range: 15–190) in non-treated communities. Demographic, socioeconomic, and environmental characteristics of the children from treated and non-treated communities are shown in Table 1. There were differences in some socioeconomic and environmental characteristics between the two study groups: treated children tended to have a lower household income and fewer material goods and a greater proportion used river water than non-treated children.

**Table 1:** Characteristics of 3,705 school-age children by ivermectin treatment group.

	No Treatment	Ivermectin	Total*	p value
	1953	1752	3705	
**Demographic characteristics**				
Age, years				
6–7	300 (15.4)	263 (15.0)	563 (15.2)	
8–9	473 (24.2)	415 (23.7)	888 (23.9)	
10–11	448 (22.9)	404 (23.0)	852 (23.0)	
12–13	453 (23.2)	376 (21.5)	829 (22.4)	
14–16	279 (14.3)	294 (16.8)	573 (15.5)	0.655
Sex, %				
Girl	983 (50.3)	827 (47.2)	1810 (48.9)	
Boy	970 (49.7)	925 (52.8)	1895 (51.1)	0.075
**Socioeconomic characteristics**				
Maternal educational level, %				
Secondary completed	156 (8.3)	132 (7.6)	288 (8.0)	
Primary completed	753 (40.1)	483 (28.0)	1236 (34.3)	
Illiterate or incomplete primary	970 (51.6)	1111 (64.4)	2081 (57.7)	0.087
Household income, %				
>150 per month	499 (25.7)	175 (10.2)	674 (18.5)	
≤150 per month	1441 (74.3)	1537 (89.8)	2978 (81.5)	<0.001
Electric appliances in house,%				
3–4	668 (34.2)	350 (20.0)	1018 (27.4)	
1–2	1066 (54.6)	963 (55.0)	2029 (54.8)	
None	219 (11.2)	439 (25.0)	658 (17.8)	<0.001
**Environmental characteristics**				
Crowding (people per sleeping room), %				
≤2	512 (26.2)	568 (32.4)	1080 (29.2)	
>2	1439 (73.8)	1183 (67.6)	2622 (70.8)	0.130
Bathroom, %				
Toilet or latrine	1081 (55.3)	1214 (69.4)	2295 (62.0)	
Open ground	872 (44.7)	536 (30.6)	1408 (38.0)	0.118
Drinking water,%				
Well	305 (15.6)	30 (1.7)	335 (9.0)	
Piped	594 (30.4)	15 (0.9)	609 (16.4)	
River or stream	1054 (54.0)	1707 (97.4)	2761 (74.6)	<0.001
**Treatment**				
Recent anthelmintic treatment, %**				
Yes	1504 (78.0)	1249 (77.5)	2753 (77.8)	
No	279 (14.5)	208 (12.9)	487 (13.8)	
Don't know	145 (7.5)	155 (9.6)	300 (8.4)	0.719

Brackets show percentages.

*: There are missing values for maternal education level (100), household income (37), crowding (3) and bathroom (2).

**: Anthelmintic treatment within the previous 6 months.

The number of doses of ivermectin received by the 1,752 children in the treated communities were: 1–5 doses (12.5%), 6–10 doses (41.3%); 11–15 doses (41.0%) and 16–20 doses (5.2%). Treatment coverage with ivermectin was high and 79.3% (1,390) of children had received >75% of designated treatments over the previous five years. Reported treatments with other anthelmintic drugs were equally widespread in both treatment (77.5%) and non-treatment (78.0%) communities over the previous 6 months. Most treatments were bought directly by parents, were distributed through schools, or through physician consultations. During the course of this study there were no programmes of systematic periodic treatments with other anthelmintic drugs such as albendazole in any of the study communities. School-based treatments with albendazole have been given sporadically by non-governmental organisations, and politically-affiliated groups (e.g. at the time of local and presidential elections every 4 years).

Community treatments may have important effects on the transmission of STHs. To investigate this, we analysed stool samples from children aged 0–5 years that had not received ivermectin. A total of 776 and 925 children from 18 treated and 18 non-treated communities, respectively, were analysed. Of the 776 children aged 0–5 years from treated communities, 484 (62.8%) had not received any dose of ivermectin (for reasons of weight <15 kg), 139 (18.0%) had received 1–2 doses, 96 (12.5%) 3–4 doses, 42 (5.4%) 5–6 doses and 10 (1.3%) 7–8 doses.

### Effect of ivermectin treatment on STH infections

**Figure 1:**
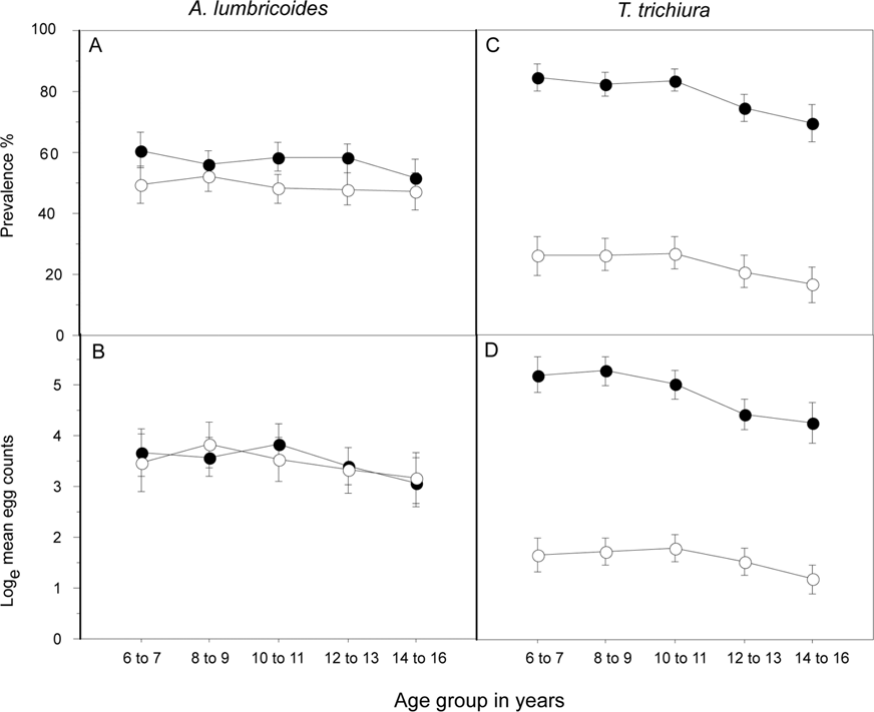
Age-prevalence and age-intensity for *Ascaris lumbricoides* (graphs A and B, respectively) and *Trichuris trichiura* (C and D, respectively) for school-age children treated (open circles) or not treated (closed circles) with ivermectin. Shown are mean values and 95% confidence intervals.

Ivermectin treatment was associated with a significant reduction in the prevalence of infection with any STH parasite (62.8% treated vs. 86.3% untreated children; adj. OR 0.27, 95% CI 0.15–0.47, P<0.001) The age-prevalence and age-infection intensity profiles for *A. lumbricoides* and *T. trichiura* infections in treated and non-treated children are shown in Figure 1. There were trends of age-associated declines in the prevalence and intensity of both infections. The prevalence (treated 48.9% vs. non-treated 57.3%, adjusted OR 0.96, 95% CI 0.45–2.05, P = 0.92) and intensity (GM infection intensity, treated 30.2 vs. untreated 33.7 epg; adjusted RR 1.51, 95% CI 0.81–2.82, P = 0.19) of *A. lumbricoides* infection was not different between children from ivermectin-treated and non-treated communities across the age groupings (Figure 1A and B) (Table 2 and 3). Both the prevalence (treated 31.1% vs. non-treated 81.5%, adjusted OR 0.06, 95% CI 0.03–0.14, P<0.001) and intensity (GM infection intensity, treated 3.9 vs. untreated 132.0 epg; adjusted RR 0.28, 95% CI 0.11–0.70, P = 0.007) of *T. trichiura* infection were greater in non-treated compared to treated school children at all age groups (Figure 1C and D) (Table 2 and 3). Ivermectin treatment did not appear to reduce the prevalence of hookworm infection and, in fact, the prevalence was significantly greater in treated compared to non-treated children (treated 14.8% vs. non-treated 3.9%, adjusted OR 5.53, 95% CI 1.81–16.86, P = 0.003) (Table 2).

**Table 2:** Impact of ivermectin treatment on STH infections in 3,705 school-age children.

	No-Treatment	Ivermectin	Total	Univariate analysis		Multivariate analysis	
	1953	1752	3705	Odds ratio	p	Odds ratio*	p
				(95% CI)		(95% CI)	
**STH infections**							
**Any helminth**							
Negative	267 (13.7)	652 (37.2)	919 (24.8)	1.0		1.0	
Positive	1686 (86.3)	1100 (62.8)	2786 (75.2)	0.24 (0.12–0.45)	<0.001	0.27 (0.15–0.47)	<0.001
***A. lumbricoides***							
Negative	834 (42.7)	895 (51.1)	1729 (46.7)	1.0		1.0	
Positive	1119 (57.3)	857 (48.9)	1976 (53.3)	0.81 (0.42–1.53)	0.512	0.96 (0.45–2.05)	0.916
***T. trichiura***							
Negative	362 (18.5)	1208 (68.9)	1570 (42.4)	1.0		1.0	
Positive	1591 (81.5)	544 (31.1)	2135 (57.6)	0.09 (0.04–0.18)	<0.001	0.06 (0.03–0.14)	<0.001
**Hookworm**							
Negative	1877 (96.1)	1492 (85.1)	3369 (90.9)	1.0		1.0	
Positive	76 (3.9)	260 (14.8)	336 (9.1)	5.71 (2.26–14.43)	<0.001	5.53 (1.81–16.86)	0.003
***S. stercoralis***							
Negative	1939 (99.3)	1750 (99.9)	3689 (99.6)	…		…	
Positive	14 (0.7)	2 (0.1)	16 (0.4)	**		**	

*: ORs adjusted for age, sex, maternal educational level, monthly income, household electric appliances, crowding, bathroom, water source and number of ivermectin treatments received.

**: Cannot be reliably estimated because of small numbers.

**Table 3:** Impact of ivermectin treatment on geometric mean infection intensities (GMI) with *A.lumbricoides* and *T.trichiura* in 3,705 school-age children.

	No-Treatment	Ivermectin	Univariate analysis		Multivariate analysis	
	1953 (52.7%)	1752 (47.3%)	RR	p	RR^*^	p
			(95% CI)		(95% CI)	
**STH infections**						
*A. lumbricoides*, GMI	33.71	30.24	1.88 (1.04–3.37)	0.036	1.51 (0.81–2.82)	0.190
*T. trichiura*, GMI	132.09	3.91	0.28 (0.18–0.43)	<0.001	0.28 (0.11–0.70)	0.007

GMI, geometric mean intensity.

Rate ratios (RR) were calculated using zero-inflated binomial regression models and adjusted for age, sex and ivermectin treatments.

Among pre-school children living in the same communities and not eligible to receive ivermectin (in treated communities), there were significantly fewer infections with any STH (untreated 64.8% vs. treated 40.7%; adjusted for age, sex, and treatment, OR, 0.34, 95% CI 0.21–0.56, P<0.001) and *T. trichiura* infection (untreated 55.4% vs. treated 15.7%; adj. OR =  0.12, 95% CI 0.07–0.21, P<0.001)) but not *A. lumbricoides* infection (untreated 38.4% vs. treated 33.1%; adjusted OR = 0.67, 95% CI 0.38–1.19, P = 0.17) among treated compared to non-treated children. Pre-school children from the treated communities also had significantly lower infection intensities with *T.trichiura* (adj. RR = 0.32, 95% CI 0.19–0.54, P<0.001) but not with *A. lumbricoides* (adj. RR = 1.51, 95% CI 0.76–3.00, P = 0.24) compared to those from non-treated communities (Table 4 and 5). Because 32.9% of children in the treated group had received at least one dose of ivermectin, the analysis was repeated excluding all treated children (925 untreated vs. 471 treated children). The results of these analyses were similar to those obtained for all pre-school children (e.g. age and sex-adjusted OR for *T. trichiura* prevalence, OR 0.11, 95% CI 0.06–0.22, P<0.001).

At the community level there was a lot of variation in the prevalence of *A. lumbricoides* between treated and non-treated communities and no evidence of systematic differences in prevalence, but for *T. trichiura* infection, there was a strong trend for reduced prevalence in treated compared to non-treated communities (data not shown).

**Table 4:** Impact of ivermectin treatment on STH infections in 1,701 pre-school children.

	No-Treatment	Ivermectin	Univariate analysis		Multivariate analysis	
	n = 925	n = 776	Odds ratio	p	Odds ratio*	p
			(95% CI)		(95% CI)	
**STH infections**						
**Any helminth,**						
Negative	326 (35.2)	460 (59.3)	1.0		1.0	
Positive	599 (64.8)	316 (40.7)	0.33 (0.22–0.50)	<0.001	0.34 (0.21–0.56)	<0.001
***A. lumbricoides***						
Negative	570 (61.6)	519 (66.9)	1.0		1.0	
Positive	355 (38.4)	257 (33.1)	0.64 (0.38–1.09)	0.102	0.67 (0.38–1.19)	0.174
***T. trichiura***						
Negative	413 (44.6)	654 (84.3)	1.0		1.0	
Positive	512 (55.4)	122 (15.7)	0.12 (0.08–0.20)	<0.001	0.12 (0.07–0.21)	<0.001
**Hookworm**						
Negative	920 (99.5)	768 (99.0)	–	…	…	…
Positive	5 (0.5)	8 (1.0)	**		**	
***S. stercoralis***						
Negative	904 (97.7)	775 (99.9)	…	…	….	…
Positive	21 (2.3)	1 (0.1)	**		**	

*: Odds ratio adjusted by age, sex and ivermectin treatment.

**: Cannot be reliably estimated because of small numbers.

**Table 5:** Impact of ivermectin treatment on geometric mean infection intensities (GMI) with *A. lumbricoides* and *T. trichiura* in 1,701 pre-school children.

	No-Treatment	Ivermectin	Univariate analysis		Multivariate analysis	
	n = 925	n = 776	RR	p	RR*	p
			(95% CI)		(95% CI)	
**STH infections**						
***A. lumbricoides,*** GMI	8.57	6.38	1.30 (0.67–2.51)	0.438	1.51 (0.76–3.00)	0.241
***T. trichiura*** *,* GMI	11.19	0.82	0.26 (0.17–0.39)	<0.001	0.32 (0.19–0.54)	<0.001
Hookworm, GMI	0.02	0.03	**		**	

*: Rate Ratios (RRs) calculated by zero-inflated negative binomial regression models and adjusted by age, sex and ivermectin treatment.

**: Cannot be reliably estimated because of small numbers.

## Discussion

The control of soil-transmitted helminth (STH) infections is an important public health strategy targeted at school-age children in many developing countries, and has been prioritised because of the important morbid effects attributed to these infections. Further, such programmes are relatively easy to administer (through schools) and are considered to be highly cost-effective [21],[22]. Because STH infections are caused by faecal contamination of the environment, re-infections may be frequent after a single dose of anthelmintic treatment in highly endemic areas [23], and because treatment programmes rarely address underlying causes (e.g. poor sanitation), treatments may have to be administered periodically for periods of years. There are few reports of the long-term effects of anthelmintic treatment programmes on the epidemiology of STH infections; many studies, both randomized controlled trials [24] and uncontrolled studies [25], have reported the effect of single doses of anthelmintic drugs either alone or in combination with follow-up of up to 1 year post treatment [10]. Few studies have investigated the impact of periodic treatments with anthelmintic drugs on STH infections for longer periods–in such studies, follow-up has ranged 2 [26] and 4 years [27].

In the present study, we measured the impact of up to 17 years of periodic treatments with the broad-spectrum anthelmintic drug, ivermectin, on both the epidemiology of STH infections in school-age children and also the impact of such treatments on the transmission of STH infections in pre-school children. Our results provide evidence that long-term ivermectin treatments may have differential effects on STH infections, with major effects on *T. trichiura*, but little or no effect on *A. lumbricoides* and hookworm infections.

The present study had several important strengths. Ivermectin is a broad-spectrum anthelmintic drug that is effective against STH infections and has comparable efficacy (except for hookworm) to albendazole, the most widely used drug in STH control programmes [10],[11],[12]. Periodic treatment in this study was given for periods of between 15 and 17 years, making it, to our knowledge, the longest documented period of periodic treatments used for the evaluation of the impact of anthelmintic treatment on STH infections. Further, the data is from an operational control programme that was able to achieve high rates of treatment coverage, and the data obtained reflect, therefore, what an optimal control programme may be capable of achieving using a strategy of twice-annual treatments. Other strengths were the availability of data on the number of ivermectin treatments at the individual level, censuses that allowed us to identify all children of school age in each community, the collection of data on important confounding factors, and the study of a large sample both at the individual and community levels.

However, the study was cross-sectional rather than prospective and we did not have data on STH infections before the start of the intervention. We tried to select comparable non-treated communities, but there was evidence of differences in some variables between the treated and non-treated communities at baseline (Table 1). Although an analytic strategy allowing for these factors to be controlled in the analysis was used, residual confounding or systematic bias cannot be excluded. Systematic differences between communities may explain the higher prevalence of hookworm observed in treated communities. This observation is unlikely to be explained by the limited of efficacy of ivermectin against hookworm infections [12],[13],[28],[29]. The determinants of the geographic distribution of hookworm and other STH infections are poorly understood but include factors such as climate, socioeconomic factors and human behaviour [30]. We do not have data on climatic variables for these communities, and there were differences between treated communities with respect to some socioeconomic indicators (e.g. median income and material goods in the household) that may explain partly the geographic distribution of hookworm infection.

The study provided evidence that long-term periodic treatments with ivermectin may have important effects on the prevalence and intensity of *T. trichiura* infections. Previous studies have demonstrated variable efficacy of ivermectin against *T. trichiura* infections after one (35%–88% cure rate) [10],[11],[13],[14] or two doses (100% cure rate) [13] although studies from Africa showed very limited efficacy after 1–4 doses of ivermectin (0–11% cure rates) [12],[29],[31],[32],[33]. The present study in which communities had received ivermectin for 15–17 years provided some evidence for strong and significant long-term effects against *T. trichiura* infections among children receiving treatment (reduction in prevalence and intensity of 50.4% and 72%, respectively). Further, the observation of a reduced *T. trichiura* infection prevalence and intensity (39.7% and 68%, respectively) among children not eligible to receive treatment suggest that long-term ivermectin treatments may suppress the transmission of this infection.

Surprisingly, we did not observe an effect of long-term ivermectin administration on *A. lumbricoides* infections. Ivermectin is extremely effective against this parasite and single dose cure rates between different studies are consistently greater than 78% [10],[11],[12],[13],[14]. Studies that have examined the effects of ivermectin against ascariasis have shown that a single dose could reduce the prevalence and intensity of *A. lumbricoides* infection for up to three months after a single treatment [29],[34], although reinfections are an important problem and may occur by 3 months after treatment [29],[31],[35]. Previous studies that have examined the effects of multiple doses of ivermectin against *A. lumbricoides* infection for periods of 1–2 years have shown no important effect of treatments on the prevalence of *A. lumbricoides* infection [31],[33]. Further, in this study we did not observe an impact of ivermectin treatment on the prevalence or intensity of infection of *A. lumbricoides* among children not eligible to receive treatment, indicating little effect on the transmission of infection.

There are three possible explanations for the possible lack of long-term effect of ivermectin on ascariasis. Firstly, fertilized *A. lumbricoides* adult females are extremely fecund and each may produce in excess of 200,000 embryonated eggs per day [36], considerably more than *T. trichiura* or hookworm females. *Ascaris* eggs are extremely resistant to adverse environmental conditions and may remain infectious for years. Thus, in the absence of adequate removal of human faeces, exposure to embryonated eggs in the environment in endemic communities may be difficult to avoid and reinfection inevitable. A city-wide intervention to provide sanitation in the city of Salvador in Brazil had dramatic effects in reducing diarrhoeal incidence [37] and the prevalence of STH infections including ascariasis [38] that have been sustained over many years. A second but perhaps less likely explanation is the development of drug resistance by *A. lumbricoides* to ivermectin. Mass treatments with ivermectin have been administered in these communities for 15–17 years and the development of drug resistance by *A. lumbricoides* could explain the lack of impact of ivermectin on this infection. Decreased sensitivity of hookworm infections to mebendazole [39] and pyrantel [40] has been reported previously, and resistance to ivermectin by gastrointestinal nematodes of animals is widespread [41]. Finally, we do not have pre-treatment data on the prevalence of ascariasis in treated communities and it is possible that the prevalence of ascariasis was higher in treated compared to non-treated communities before the start of mass treatment with ivermectin between 1990 and 1992. Data obtained from the pre-treatment period for a group of 158 adults and children from a treated Afro-Ecuadorian community, not included in the present study, indicated a prevalence of *A. lumbricoides* and *T. trichiura* infections of 58.2% and 59.5%, respectively [17]. Although such data should be interpreted cautiously–because of differences in age, study population, and the diagnostic method used - they may suggest that the prevalence of ascariasis has not altered greatly since the start of mass treatment and that long-term ivermectin treatments have had a relatively greater effect in reducing the prevalence of trichuriasis than ascariasis.

### Conclusions

We have evaluated the impact of long-term treatment with a broad-spectrum anthelmintic drug, ivermectin, used for the control of onchocerciasis, on the epidemiology of STH infections in rural Ecuador. The data indicate that 15–17 years of annual or twice-annual ivermectin treatments was highly effective against *T.trichiura* infections but may have had little impact on infections with *A. lumbricoides* and hookworm. Our study provides evidence that control programmes using ivermectin may provide additional health benefits by reducing the prevalence and intensity of infections with trichuriasis. To have a greater impact on ascariasis and hookworm infections, such programmes could consider the addition of twice annual albendazole treatments that can be safely administered with ivermectin [25], although effective reductions in the prevalence of these infections may require more frequent periodic treatments. The combination of ivermectin and albendazole has the advantage also of greater efficacy against trichuriasis than either drug alone [10],[11],[42]. However, long-term sustainable control may require interventions that target the underlying causes of these infections–namely the unsafe disposal and use of human faeces.

## Supporting Information

Alternative Language Abstract S1 Translation of the Abstract into Portuguese by Mauricio Barreto
(0.02 MB DOC)

Alternative Language Abstract S2 Translation of the Abstract into Spanish by Ana Lucia Moncayo
(0.02 MB DOC)
