# Supplementary material for: Impact of Long-Term Treatment with Ivermectin on the Prevalence and Intensity of Soil-Transmitted Helminth Infections
Source: PLoS Negl Trop Dis. 2008 Sep 10;2(9):e293. doi: 10.1371/journal.pntd.0000293 (PMC2553482; doi:10.1371/journal.pntd.0000293)
Supplement: Alternative Language Abstract S1 — Translation of the Abstract into Portuguese by Mauricio Barreto (0.02 MB DOC) [file pntd.0000293.s001.doc]

**Resumo**

**Introdução:** O controle de infecções causadas por geohelmintos esta baseado na administração regular e de longa duração de drogas anti-helmínticas a grupos de alto risco, particularmente as crianças em idade escolar que vivem em áreas endêmicas. Existem dados limitados sobre a efetividade dos tratamentos anti-helmínticos periódicos a longo prazo na prevalência de infecções com geohelmintos, particularmente a partir de programas operacionais. Este estudo investigou o impacto do tratamento em massa por 15 a 17 anos com um anti-helmíntico de amplo espectro, ivermectina, usada para controlar oncocercosis, na prevalência e na gravidade da infecção por geohelmintos em escolares e pré-escolares.

**Métodos:** Um estudo transversal foi realizado em comunidades que haviam recebido tratamento anual ou semestral com ivermectina e comunidades adjacentes que não haviam recebido esse tratamento em dois cantões da Província de Esmeraldas no Equador. Foram colhidas amostras de fezes de escolares que foram analisados para infecção por geohelmintos utilizando-se técnicas de Kato Katz e de concentração em formol-éter. Amostras de fezes foram também coletadas de pré-escolares e examinadas por técnica de concentração com formol-éter. Dados sobre os fatores de risco para infecções por geohelmintos foram coletados através de questionários aplicados aos pais.

**Resultados**: Foram estudadas um total de 3705 crianças em idade escolar (6-16 anos) de 31 comunidades tratadas e 27 não tratadas e 1701 pré-escolares com idade de 0 a 5 anos em 18 comunidades tratadas e 18 não tratadas. No que diz respeito aos escolares, o tratamento com ivermectina teve efeitos significativos sobre a prevalência (OR ajustado = 0,06, 95% IC 0,03-0,14) e na intensidade da infecção *Trichuris trichiura* (RR ajustado = 0,28, 95% IC 0,11 -- 0,70), mas não mostrou qualquer impacto sobre a infecção por *Ascaris lumbricoides* ou ancilostomíase. A redução da prevalência e intensidade de infecção por *T. trichiura* foi observada em crianças não elegíveis para receber ivermectina, fornecendo evidência de que houve redução na transmissão da infecção por *T.trichiura* nas comunidades que haviam recebido tratamento em massa com ivermectina.

Conclusão: O tratamento com ivermectina anual ou semestral, por um período de cerca de 17 anos tiveram um impacto significativo sobre a infecção por *T. trichiura*. Os dados apresentados neste estudo indicam que a longo prazo o controle da oncocercosis com ivermectina pode proporcionar benefícios adicionais da saúde através da redução das infecções por *T. trichiura*. A adição de uma segunda droga anti-helmíntica como albendazol, seria útil para conseguir um efeito a longo prazo a infecção A. lumbricoides.

**Palavras-chave:** ivermectina, geohelmintos, oncocercosis, crianças em idade escolar, Equador.
