# Supplementary material for: Impact of Long-Term Treatment with Ivermectin on the Prevalence and Intensity of Soil-Transmitted Helminth Infections
Source: PLoS Negl Trop Dis. 2008 Sep 10;2(9):e293. doi: 10.1371/journal.pntd.0000293 (PMC2553482; doi:10.1371/journal.pntd.0000293)
Supplement: Alternative Language Abstract S2 — Translation of the Abstract into Spanish by Ana Lucia Moncayo (0.02 MB DOC) [file pntd.0000293.s002.doc]

**RESUMEN**

**Introducción:** El control de las infecciones causadas por geohelmintos está basado en la administración periódica y a largo plazo de drogas antihelmínticas a los grupos de alto riesgo, particularmente a niños en edad escolar que viven en áreas endémicas. Existen datos limitados sobre la efectividad de tratamientos antihelmínticos periódicos a largo plazo en la prevalencia de las infecciones por geohelmintos particularmente desde programas operacionales. El presente estudio investigó el impacto de 15 a 17 años de tratamiento en masa con un antihelmíntico de amplio espectro, la ivermectina, usada para el control de la oncocercosis, en la prevalencia e intensidad de infección por geohelmintos en niños escolares y pre-escolares.

**Métodos:** Un estudio transversal fue conducido en comunidades que han recibido tratamientos anuales o bianuales con ivermectina y en comunidades adyacentes que no han recibido este tratamiento, en dos cantones de la Provincia de Esmeraldas en Ecuador. Muestras de heces fueron colectadas de los niños escolares y fueron examinadas para la infección por geohelmintos usando las técnicas de Kato Katz y concentración con formol-éter. Muestras de heces también fueron colectadas de los niños pre-escolares y examinadas por la técnica de concentración con formol-éter. Datos sobre los factores de riesgo para las infecciones por geohelmintos fueron obtenidos por medio de cuestionarios aplicados a los padres.

**Resultados:** Fueron investigados un total de 3,705 niños escolares (6-16 años) de 31 comunidades tratadas y 27 comunidades no tratadas y 1,701 pre-escolares con edades entre 0 y 5 años de 18 comunidades tratadas y 18 no tratadas. Con respecto a los niños escolares, el tratamiento con ivermectina tuvo efectos significantes en la prevalencia (OR ajustado= 0.06, 95% IC 0.03-0.14) y en la intensidad de infección por *Trichuris trichiura* (RR ajustado=0.28, 95% IC 0.11-0.70), pero no mostró tener un impacto en la infección por *Ascaris lumbricoides* o uncinarias. Una reducción en la prevalencia y en la intensidad de infección por *T. trichiura* fue observada en los niños no elegibles para recibir ivermectina brindando alguna evidencia de una reducción de la transmisión de la infección por *T.trichiura* en las comunidades que han recibido tratamientos en masa con ivermectina.

**Conclusión:** Tratamientos con ivermectina anuales y bianuales por un periodo de alrededor de 17 años han tenido un significante impacto en la infección por *T. trichiura*. Los datos presentados en este estudio indican que el control a largo plazo de la oncocercosis con ivermectina brindaría beneficios adicionales para la salud a través de la reducción de las infecciones con trichiurasis. La adición de una segunda droga antihelmíntica, tal como el albendazol, sería útil para conseguir un efecto a largo plazo en la infección por *A. lumbricoides*.

Palabras clave: ivermectina, geohelmintos, oncocercosis, niños escolares, Ecuador.
